# Michigan Veterinary Medical Association

**Published:** 1901-04

**Authors:** W. A. Giffen

**Affiliations:** Secretary


### SOCIETY PROCEEDINGS.

### MICHIGAN VETERINARY MEDICAL ASSOCIATION.

The nineteenth annual meeting was held in Lansing, February 5 and 6, 1901, President William Jopling presiding. Thirteen new members were added to the roll, making a total of over eighty members in good standing.

The reports of all the committees were very thorough, showing that the work had been done earnestly and carefully. The results of the meeting showed that the members of the profession were working together harmoniously, not only among themselves, but with the State Board of Health, and the State Live-Stock Sanitary Commission.

The roll-call showed forty-five members present, and a number of the members brought their ladies with them, for whom a good programme of entertainments was arranged and a banquet was provided for the evening of February 5th, in which the ladies participated, Dr. H. F. Palmer presiding as toast master. The Treasurer's report showed a balance in the treasury of $204.21.

Papers read and discussed were as follows: "The Veterinarian as a Sanitary Officer," by Dr. James Drury; "Methods of Inspecting Dairies," by Dr. Charles E. Marshall; "Ovariotomy Bovidæs," by Dr. H. S. Smith; "Treatment of Wounds," by Dr. W. S. Hamilton; "Infection," by Dr. George A. Waterman; "A Contagious Disease Affecting the Eyes of Cattle," by Dr. W. M. Burdick; "The Veterinarian as a Municipal Officer," by Dr. J. A. Dell; "Benign Growths," by Dr. H. F. Palmer; "Parturient Apoplexy and Its Treatment," by Dr. H. M. Gohn; "Healthful Legislation," by Dr. H. B. Barker; "The Veterinarian in Politics," by Dr. G. W. Dunphy; "Strongylus Contortus" and "Strongylus Ovis Pulmonalis" and "Successful Treatment by the Use of Toxaline," by Dr. J. J. Walkington.

Officers elected and committees appointed for the ensuing year as follows: President, J. J. Joy, Detroit; First Vice-President, H. F. Palmer, Detroit; Second Vice-President, H. M. Gohn, St. Johns; Third Vice-President, James Harrison, Maple Rapids; Secretary and Treasurer, W. A. Giffen, Detroit.

Committee on Intelligence and Education: H. F. Palmer, Detroit, Chairman; George A. Waterman, Lansing; U. S. Springer, Grand Rapids. Diseases: H. M. Gohn, St. Johns, Chairman; G. W. Dunphy, Quincy; Thomas Farmer, Grand Blanc. Finances: W. S. Hamilton, Chelsea, Chairman; J. C. Whitney, Hillsdale; James Irving, Ypsilanti. Legislation: W. A. Giffen, Detroit, Chairman; F. C. Wells, Warren; J. Black, Richmond; G. W. Dunphy, Quincy. Directors elected: J. Black, Richmond; J. W. Brodie, Pontiac; D. G. Sunderland, Saginac; H. S. Smith, Albion; J. J. Walkington, Mt. Pleasant; A. McKercher, Lansing.

W. A. Giffen,
Secretary.

